# Toward FRP-Based Brain-Machine Interfaces—Single-Trial Classification of Fixation-Related Potentials

**DOI:** 10.1371/journal.pone.0146848

**Published:** 2016-01-26

**Authors:** Andrea Finke, Kai Essig, Giuseppe Marchioro, Helge Ritter

**Affiliations:** 1 Center of Excellence Cognitive Interaction Technology CITEC, Bielefeld University, Bielefeld, Germany; 2 Neuroinformatics Group, Technical Faculty, Bielefeld University, Bielefeld, Germany; 3 Neurocognition and Action Group, Faculty of Psychology, Bielefeld University, Bielefeld, Germany; 4 Department of Computer Science, Verona University, Verona, Italy; University of Electronic Science and Technology of China, CHINA

## Abstract

The co-registration of eye tracking and electroencephalography provides a holistic measure of ongoing cognitive processes. Recently, *fixation-related potentials* have been introduced to quantify the neural activity in such bi-modal recordings. Fixation-related potentials are time-locked to fixation onsets, just like event-related potentials are locked to stimulus onsets. Compared to existing electroencephalography-based brain-machine interfaces that depend on visual stimuli, fixation-related potentials have the advantages that they can be used in free, unconstrained viewing conditions and can also be classified on a *single-trial* level. Thus, fixation-related potentials have the potential to allow for conceptually different brain-machine interfaces that directly interpret cortical activity related to the visual processing of specific objects. However, existing research has investigated fixation-related potentials only with very restricted and highly unnatural stimuli in simple search tasks while participant’s body movements were restricted. We present a study where we relieved many of these restrictions while retaining some control by using a gaze-contingent visual search task. In our study, participants had to find a target object out of 12 complex and everyday objects presented on a screen while the electrical activity of the brain and eye movements were recorded simultaneously. Our results show that our proposed method for the classification of fixation-related potentials can clearly discriminate between fixations on relevant, non-relevant and background areas. Furthermore, we show that our classification approach generalizes not only to different test sets from the same participant, but also across participants. These results promise to open novel avenues for exploiting fixation-related potentials in electroencephalography-based brain-machine interfaces and thus providing a novel means for intuitive human-machine interaction.

## Introduction

Brain-machine interfaces (BMI) based on the electroencephalogram (EEG) have been around for more than two decades now. Various approaches, defined by the particular EEG component they exploit, have been developed and tested. Among the two most prominent components are the P300 event-related potential (ERP) and steady-state visually evoked potentials (SSVEP). Both are elicited by visual stimuli. The P300 or P3 ERP is triggered by a rare target that occurs in a sequence of frequent non-targets—a structure called oddball paradigm. BMI systems usually implement the oddball paradigm by sequentially highlighting symbols or icons on the computer screen in a random sequence for a short period of time (e.g., 100 ms). The flashing of target symbols will elicit a P300 if they are relevant for a particular user or a given task, while the other symbols will not. One famous implementation of a P300-based BMI is the P300 Speller, a system where rows and columns of characters are highlighted in random order, e.g., [1], [2]. SSVEPs are elicited by stimuli flickering at a particular frequency [3]. They either use stimuli on a computer screen (e.g., a checkerboard-style pattern as in [4]) or dedicated hardware devices with LEDs (e.g., [5]). SSVEP-based BMI systems are usually rather fast and reliable, due to the simple, frequency-based structure of the cortical response and the option to flicker several stimuli simultaneously [6]. Recently, also BMIs based on codebook visually evoked potentials (cVEP) are becoming increasingly popular [7]. In contrast to SSVEPs, the neural responses to stimuli in cVEPs depend non-linearly on flickering patterns specified by binary codebook vectors. Please refer to Bin et al. [8] for a concise comparison of SSVEPs and cVEPs for BMI.

SSVEPSs and cVEPs are both not capable of extracting information about the relevance of scene objects without additional visual augmentation (e.g., overlaying a scene with flickering stimuli). Additionally, there is no direct correspondence between the neural activation and object relevance. This correspondence is only indirectly established via the experimental paradigm, particularly for the VEP: Consider a set of stimuli flickering at different frequencies attached to objects. Then, the neural response corresponding to the selection of an object is a low-level response of the primary visual cortex (V1) to the particular flickering frequency. Identifying and selecting an object, however, is a high-level cognitive task, involving the full neural activation hierarchy from the processing of low-level visual features up to a full semantic understanding of the identified object and its relevance for a particular task.

In order to overcome the limitations of existing EEG-based BMI systems described above, we present a novel and conceptually different approach based on fixation-related potentials (FRPs). FRPs (together with the early ERP components) constitute a complex activation pattern that particularly reflects the high-level object and task identification processes. Based on spatial and temporal information on user’s gaze movements provided by the eye tracker, our system will respond only when objects are relevant to the user and/or the tasks at hand.

In this regard, FRP is similar to the P300 component (please refer to [9] for the cognitive and neural foundations), but the latter one cannot be used without a stimulus presentation respecting the oddball paradigm. Consequently, it is not applicable for free scene exploration (as this would require to flash the objects in the scene in a random sequence) and the real-time decoding of P300 potentials in complex real-world scenarios is highly limited. Consequently, analyzing EEG and eye tracking data simultaneously (i.e., aligning EEG epochs to fixation onsets) allows for free scene exploration and the detection of relevant objects becomes feasible. This may provide a novel means for natural and intuitive BMI interfaces for real world explorations.

The remaining paper is structured as follows. First, we introduce the theoretical considerations that our approach draws on. Then, we describe the details of our study followed by a section on the methods that we applied for data analysis and classification. We present the results of our study and discuss them before drawing a conclusion.

### Theoretical Foundations

#### Fixation Related Potentials

While eye tracking provides indirect measures (for example, fixation durations) from which conclusions on the underlying cognitive processes can be drawn [10] it does not give insights into ongoing brain processes. By simultaneously recording eye tracking and EEG data the neural activation during scene exploration can be investigated in order to get a rather holistic picture of cognitive processing. In case of a visual search task, for instance, eye tracking data alone is insufficient to reliably identify when a participant found the target. Fixation duration could be an indicator, but several competing reasons can explain longer fixation times on particular objects (e.g., problems with object identification or positive associations with the object). Additionally, people may identify target objects quite fast with a quick glance on them.

Fixation-related potentials (FRP) are averaged potentials aligned to fixation onsets (like ERPs, which are locked to stimulus onset). FRPs have similar structural properties as the P300 potential, that is, latency, amplitude and morphology [11]. The P300 potential is one of the best studied event-related potentials. The P300 is not one single component, although the common BCI literature treats is that way. It subsumes the P3a and the P3b (the “BCI-P300” is the P3b). Furthermore, it has not yet been possible to link the P300 to one single cognitive process, but it seems to reflect “a culmination of multiple cognitive processes” [12]. Despite not being elicited in an oddball task here, FRP potentials may add a further element or component to the P300 complex: In case of FRPs, the fixation onset substitutes for the missing temporal marker, the trigger, that indicates the onset of a stimulus and is essential for EEG data segmentation. Consequently, the eye tracking data provides the missing information that is needed to infer the selection of a target from a participant’s EEG data. This information comprises the fixation location, temporal onset and duration. Consequently, the simultaneous and synchronized acquisition, processing and classification of EEG and eye tracking allows for brain interfaces that can detect relevant events (e.g., the identification of a target object during unconstrained and natural scene exploration.

#### Related Work

The authors in [13] were the first who studied concurrent eye movements and electroencephalographic (EEG) recordings in a visual search task for hidden targets in an array. Their FRP sequence highly resembles the ERPs in a replay experiment. The participants either kept fixation while a sequence of images occurred around the fovea simulating the spatial and temporal patterns during the free viewing experiment, or where experimenters controlled the appearance of the stimuli on the screen center. Woldorff [14] also applied eye fixations to generate ERPs during a visual search task. They observed clear differences in ERPs following training, suggesting that neurophysiological signatures could be developed to prevent errors in visual search tasks. The authors in [11] used FRPs to differentiate between target and non-target fixations. Their targets consisted of the letter “C” rotated in steps of 90°. The work described in [15] demonstrated the applicability of simultaneous EEG and eye movement recordings in two reading conditions, text-reading and a pseudo-reading control condition. They found effects of the reading condition in early ERP components. Furthermore, they indicated that the co-registration of eye tracking and EEG has the potential to become an important tool for investigating the cognitive and neural bases of on-line language processing in reading. Their classification analysis was based on response patterns of individual observers, because these patterns may not be visible in overall FRP averages.

Lao et al. [16] conducted an EEG study on the interaction between culture and attention sensitivity to global/local information processing in Navon figures. These figures are hierarchical stimuli comprising a large global shape constituted by small local shapes (e.g. a global T composed of smaller letters S) making them suitable to investigate the preferences to either local or global elements [17]. East Asians showed differential electrophysiological responses in the P1 component paired with a more efficient processing of global feature changes at later stages. In contrast, Western Caucasians showed preferences to their preferred (generally slower) local feature information coding at later (200–350ms), but not in the early component. While East Asians performed equally well while detecting global and local features changes, Westerners were more efficient at detecting local changes [16]. Therefore, East Asians seemed to benefit from a top-down attention control to global features and were less disturbed by global/local visual saliency feature differences than Westerners, who showed an attention bias toward local features.

#### Combining EEG and Eye Tracking Recordings

**Fig 1:**
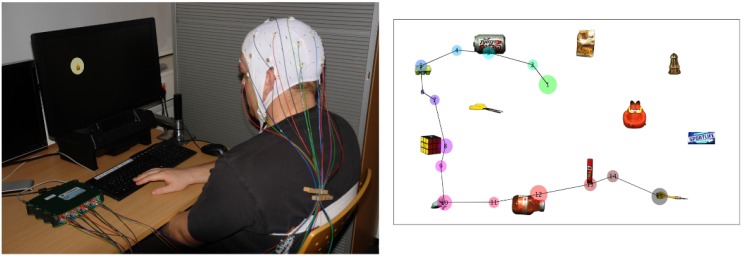
**Left:** A participant is performing the search task while his eye movements and EEG data are recorded simultaneously. The black box below the screen is the EyeFollower remote eye tracker. **Right:** Example scanpath of participant 1 who has to find the dart out of 12 objects in trial 48. The circles denote fixations, where the size is proportional to the fixation duration and the numbers represent their chronological order. The lines symbolize saccades. The Figure illustrates that participants apply a grid structure like scanpath to successively check the different objects for a match in the gaze-contingent search task.

The downside of the FRP approach is that eye movements introduce technical and analytical challenges/ artifacts on the EEG recordings#x2014;a reason why the simultaneous acquisition of EEG and eye-tracking data has rarely been applied. These artifacts do not only emerge from different independent sources, but also these sources contribute differently to the measured signal (depending on electrode site, gaze direction, and choice of reference) [18]. These artifacts are usually reviewed individually and therefore algorithm filter solutions are only accurate at a particular artifact while the relative contributions of others are generally over—or under-corrected. The avoidance, identification, characterization and correction of eye-movement artifacts in EEG data is not a trivial task. In the following we describe the three most important challenges for simultaneous recording of EEG and eye-tracking data and how we will circumvent them:

1. **Precise co-registration of gaze position**: The contact between an eye tracker and EEG electrodes usually induces electromagnetic artifacts, such as the 50 Hz line noise. We circumvent this issue by using a remote eye tracker with dynamic cameras and a careful adaption of the participants sitting position (see Fig 1, right). This allows for the recording of high resolution gaze data without obstructing EEG recordings, while maintaining participants’ freedom of slight body movements.
2. **Eye movement artifacts**: There are many algorithms which effectively remove artifacts whose respective sources are well defined and whose spectral and statistical signal properties (e.g. amplitude, variance, frequency range, curtosis, as well as signal drifts caused by changing electrode properties, line noise and high frequency muscle activity) differ considerably from those of neural activity ([18, 19]). The widely applied Independent Component Analysis (ICA) can also make use of gaze data to objectively remove eye-artifact related ICA-components. The relevant signals from neural sources are kept intact [20].
3. **Differential overlap**: It is important to ensure that target fixations do not differ in terms of overlapping background activity. This problem can be solved by excluding early fixations and by guaranteeing that the pre-fixation baseline activity does not differ. Additionally, bottom-up features (such as luminance or contrast) of fixated regions, as well as different saccade amplitudes, cause relevant FRP modulations. These artifacts can be reduced by covering the peripheral field with a mask or by systematically investigating the modulations in the EEG signal for different fixation locations.

Before this section concludes with the aims of our study, we explain the gaze-contingent technique used in our experiments in the following paragraph.

#### A gaze-contingent search task for FRP detection

Modern eye trackers provide online access to gaze positions, allowing either to change the display content or to initiate an action depending on where or what a participant is looking at. In (inter-)active gaze contingency the participant is actively and consciously controlling a user interface (e.g., by initiating a corresponding action through button selection). In contrast, in passive gaze contingency, participants are neither required to actively control the appearance of the display nor are they consciously aware of the contingent display changes (e.g., sharpening the focal information while blurring the periphery). The display content is updated during a saccade so that the changes are completed before the visual intake begins with the next fixation [10]. High accuracy and sampling frequency, a low and constant latency and tight connections between recording system and stimulus presentation are the prerequisites for eye-tracking systems to allow for gaze contingency. The gaze contingent window technique provides powerful experimental control and has long been used in reading, scene perception, visual search and psycholinguistic research (see [21, 22] for a review). In the study described in [23] for example, only the letters within the contingent controlled moving window were legible, whereas those outside of the windows were either blurred, darkened or masked. This technique allowed to investigate the visual span in reading by varying the window size and determine the smallest one that allows participants to read with normal speed. Pomplun et al. [24] employed the gaze-contingent window paradigm to investigate parafoveal and peripheral cueing and masking effects on saccadic selectivity in a triple-conjunction visual search task. Their item features varied along three dimensions—color, shape, and orientation. In their experiments the target and distractors shared one (color, shape, or orientation) or two features (color-shape, color-orientation, or shape-orientation), respectively. Cueing a particular feature (or feature pair) biased saccadic selectivity toward it, while masking generally reduced saccadic selectivity. These findings support the concept of visual guidance being a pre-attentive process that operates in parallel across the display [11, 25]. In sport science studies the visual occlusion technique is used to hide body parts or movements, by image, video or gaze contingent editing. This allows for investigating how participants can anticipate the best course of action depending on their level of expertise (perceptional-cognitive skills) [26]. All in all, it can be seen that gaze contingency has long been used in research on vision, reading, psycholinguistics and sports [10]. It provided researchers many insights into the underlying cognitive processing in reading and scene perception, e.g. on visual search, the perceptual span in reading, the nature of central vs. peripheral perception, as well as the relative influence of attention versus visual acuity drop-off in the perceptual span [23, 24, 27]. By changing the size of the keyhole, the amount of perceived information can be easily controlled by the experimenter. In our experiments we used a keyhole size which is smaller than the distance between the twelve individual objects comprising one stimulus. This ensures that the participants can only perceive one object at time.

### Aim of the present study

All in all, existing research has investigated FRPs only with very restricted and highly unnatural stimuli in simple search tasks. In this paper we present a study where we relieved many of these restrictions while retaining some control by using a gaze-contingent search task with complex and everyday objects. Gaze contingency provides powerful experimental control by covering the peripheral visual field with a mask. This ensures that artifacts do not cause relevant FRP modulations due to overlapping background activity (differential overlap). Furthermore, this is beneficial for studying FRPs, because the occurrence of a potential can be exactly attributed to the relevant visual information such as a specific fixated object. Our aims is to extend and improve the existing body of research by investigating two key hypothesis/questions. Firstly, we are interested to analyze FRPs with more complex and natural stimuli. To this end, we apply natural stimuli, that is, every-day objects, together with the gaze-contingent window technique that was introduced in the previous section. Secondly, we hypothesize that the single-trial classification of FRP data can greatly benefit from applying machine learning methods that have proved successful in P300-BMI applications [2, 28]. If so, the classification accuracy should clearly improve. The following sections introduce the experiment, the methods used for classification and analysis, and present and discuss the obtained results.

## Materials and Methods

### Participants

Ten volunteers participated in the study. The participants were aged between 21 and 34 (mean 26.8 ±3.7, 7 female). All were recruited from the local student and staff population and either paid for their expenditure of time or granted course credit. All participants had no known prior or current pathological neurological condition (based on self report) and normal or corrected-to-normal vision. The experimental procedure and written consent form for this study were approved by the ethics committee at Bielefeld University, and adhered to the ethical standards of the sixth revision of the Declaration of Helsinki. All participants gave their informed written consent to participate in the study.

### Apparatus

#### Eye tracker

We used the EyeFollower (LC Technology, Clearwater, USA) remote binocular eye-tracking system for the proposed study. It allows for head movements (76 x 51 x 40cm) without the need to wear a headset. The EyeFollower has a sampling rate of 120 Hz and an accuracy of <0.4°over the whole range of head movements (see Fig 1, left).

#### EEG

We used the g.USBamp 16-channel EEG amplifier (Guger Techologies, Graz, Austria) for the study. Twelve EEG channels were recorded at the locations Fz, F3, F4, Cz, C3, C4, Pz, P3, P4, PO7, PO8 and Oz, referenced to the mastoids. Impedances were kept below 5 kΩ. Additionally, we recorded the vertical and horizontal electrooculogram (VEOG and HEOG) to register eye movements together with the EEG channels. Two VEOG electrodes were placed above and below the right eye and two HEOG electrodes beside the left and the right eye, respectively. These electrodes register the corneo-retinal standing potential that exists between the front and the back of the human eye. This data allows to investigate the influence of eye movements on the recorded EEG data. The EOG comprises four additional channels that are recorded alongside the EEG channels leading to a total of sixteen channels that are registered by the EEG amplifier.

### Stimuli

The images containing every-day objects were taken from the Amsterdam Library of Object Images (ALOI, http://aloi.science.uva.nl). An overall of 112 objects was selected, which equals the number of trials. The originally black background was converted to white. For each stimulus, twelve images were arranged in a grid-like fashion (plus random offset, see Fig 1 (right). One out of the 112 objects was selected as the target for a trial such that each object served only once as target. The remaining eleven objects per stimulus were chosen randomly from the set.

### Procedure

The participants were instructed to search for the target object and to press a key on the keyboard when they found it. Each trial started with the presentation of the target object in the center of the screen. The participants had to press a key to start the search task. We implemented a gaze-contingent approach, i.e., not the whole stimulus image was visible but only a circle area, a “keyhole”, centered at the current gaze coordinates on the screen as delivered by the eye tracker with a size of 3.5°of visual angle. The remaining part of the screen was black. The size of the visible area was chosen to cover the foveal (ca. 2°visual angle) plus half of the parafoveal area (ca. 3°). The latter corresponds to the part of the parafoveal area that still provides rather sharp vision (given the decreasing degree of sharpness toward the edges of the parafoveal area).

### Data Analysis

All algorithms for analysis, feature extraction and classification were implemented in MATLAB (Release 2015a).

#### Fixation Detection

Fixation detection on the raw eye tracking data was done using an threshold-based algorithm proposed by the manufacturer of the eye tracking device (LC Technology International Inc., Clearwater, USA. http://www.eyegaze.com/). The threshold is based on a spatial criterion, that is, consecutive samples are considered to be part of a fixation when they fall inside a circle with a diameter of 2°visual angle. Saccades were not analyzed in this study. The resulting fixations were grouped according to their locations on the stimulus image: on a target object, on a non-target object or on the blank background.

The fixation durations in the three groups did not significantly differ: Fixations on target objects had a duration of 270 ms (±38 ms) on average, non-target fixations 281 ms (±37 ms) and background fixations 241 ms (±29 ms).

The EEG data was segmented according to fixation onsets and grouped according to their locations. A fixation was considered to be an onto-object fixation, when it fell inside a circular area of 2.5°visual angle (ca. 100 pixel) anchored at the object center. Fig 1 (right) shows an example scanpath for one trial, that is, the sequence of the participant’s fixations and saccades while he/she searches for the target object.

#### Removing eye artifacts with ICA

Independent component analysis (ICA) is a blind source separation method to split a multivariate signal into linearly independent sources [20]. These sources must be linearly mixed in the recorded signal. With respect to EEG data, the sources computed by the ICA can be directly interpreted as cortical sources of neural activity that provide a unique contribution to the overall signal that is picked up. This is of particular interest because of the poor spatial distribution of the EEG method, caused by the spatial low-pass filtering effect of volume conduction. For eye movement related signal portions this effect is particularly severe because of their very high amplitudes compared to genuine cortical signals. ICA is capable of identifying eye movement related signal portions—eye artifacts—as single sources. Hence, these deteriorating artifacts can be removed by ICA. A drawback of the method is that it usually requires a human expert to identify the affected components by visual inspection. The selection is based on a component’s spatial distribution (scalp maps) and its spectral properties. In the present offline study, we used this manual selection technique to identify to-be-removed components. The ICA was computed using the EEGLAB toolbox [29], which implements an Infomax approach.

A manual selection of components is of course not possible during online operation of a system. However, the computation of the ICA projection matrix, the inspection process and the selection could be done on training data, which is needed for the classifier anyway. The stored ICA matrix with the affected components removed can subsequently be used during the online run to remove eye artifacts in an automated fashion. Moreover, Winkler and colleagues [30] have recently developed an algorithm for an automated classification of artifactual ICA components.

#### Feature Extraction and Classification

First, the continuous, multi-channel EEG data was segmented according to the temporal onsets of the fixations. Each epoch started at the the first sample of a fixation and lasted for 800 ms, which equals 205 samples per channel. The channel-wise epochs were first bandpass filtered with cut-off frequencies of 0.5 to 10 Hz. Afterwards, the channel-wise epochs were concatenated to form one high-dimensional vector $x_{i}\in R^{2460}$. Then, we applied Principal Component Analysis (PCA) to reduce the dimensionality [31]. PCA seeks to find a new set of basis vectors that represent the directions of the largest variances in the high-dimensional input space. This is achieved by determining the eigenvectors of the data covariance matrix. The corresponding eigenvalues quantify the amount of variance that is represented by the respective eigenvector. The dimensionality reduction is achieved by projecting the data into a feature space spanned by those *k* eigenvectors that represent a certain amount of variance. We used a criterion of 99.9% of variance to determine the dimensionality of the feature space. The latter is calculated by $\frac{\sum_{i=1}^{k}\lambda_{i}}{\sum_{j=1}^{n}\lambda_{j}}$ with *k* << *n* given the eigenvalues λ are sorted in descending order. Typically, this resulted in a feature space with a dimensionality around 240.

Classification was achieved using Fisher’s Linear Discriminant Analysis (FDA) [32]. The FDA is mostly used as a binary classifier separating two classes. However, in general the FDA can be applied to *n* classes using the same optimization criteria. Because the FDA is a supervised method, we need to group the feature vectors according to the class labels first resulting in *n* groups of vectors {***x***_*j*_}_*C*_*i*__ with *i* being the respective class and *j* = 1, 2, …, *N*_*C*_*i*__ where *N*_*C*−*i*_ is the number of vectors in the respective class. Then, we define the *between-class* scatter matrix as

$$S_{b}=\sum_{C_{j}}N_{c}\left(\mu_{C_{j}}-\mu \right){\left(\mu_{C_{j}}-\mu \right)}^{T},$$ (1)

with $\mu =\frac{1}{n}\sum_{C_{j}}\mu_{C_{j}}$. The *within-class* scatter matrix is given as

$$S_{w}=\sum_{i\in C_{j}}\left(x_{i}-\mu_{C_{j}}\right){\left(x_{i}-\mu_{C_{j}}\right)}^{T}$$

In practice, an adequate estimation of the scatter or covariance matrices is often difficult. This difficulty results from a disproportion of available training samples to feature dimensions. As training data is often costly, many applications suffer from too small training set sizes. This is particularly true for EEG-based BMI systems, where in addition the feature vectors extracted from the raw data are usually rather high-dimensional. To tackle this issue, Ledoit and Wolf [33] have developed a method known as the Ledoit-Wolf theorem which helps to estimate well-conditioned covariance matrices despite the described disproportion. The authors in [34] give a more practical formulation of the theorem. We have followed this approach for all covariance matrices computed during our analysis and used a shrinkage estimate instead of the pure sample covariance matrix:

$$\Sigma^{*}=\lambda \tilde{\Sigma }+\left(1-\lambda \right)\Sigma ,$$ (2)

with **Σ** being the sample covariance matrix, $\tilde{\Sigma }$ the sample covariance of a sub-model, and λ ∈ [0, 1] denotes the shrinkage intensity. As proposed in Schaefer and Strimmer [34], we use a diagonal matrix where all diagonal elements are equal, that is, all variances are equal and all covariances are zero.

Using Eqs (1) and (2), the FDA projection matrix can now be defined as the following optimization problem:

$$P_{fda}=\underset{P\in R^{d\times (n-1)}}{\mathrm{argmax}} [\mathrm{tr}\frac{P^{T}S_{b}P}{P^{T}S_{w}P}].$$ (3)

Note that this formulation is equivalent to the Rayleigh coefficient. The solution can be expressed as a generalized eigenvalue problem of the form

$$S_{b}V=\lambda S_{w}V.$$ (4)

Obviously, in case of a binary classification task, ***P***_*fda*_ reduces to a weight vector *w* and all projections are simple scalar products $y_{i}=x\cdot w^{T}+b$ and the class labels $\hat{y}\in \left\{-1,1\right\}$ can be simply determined by the sign function.

#### Performance Measure

**Fig 2:**
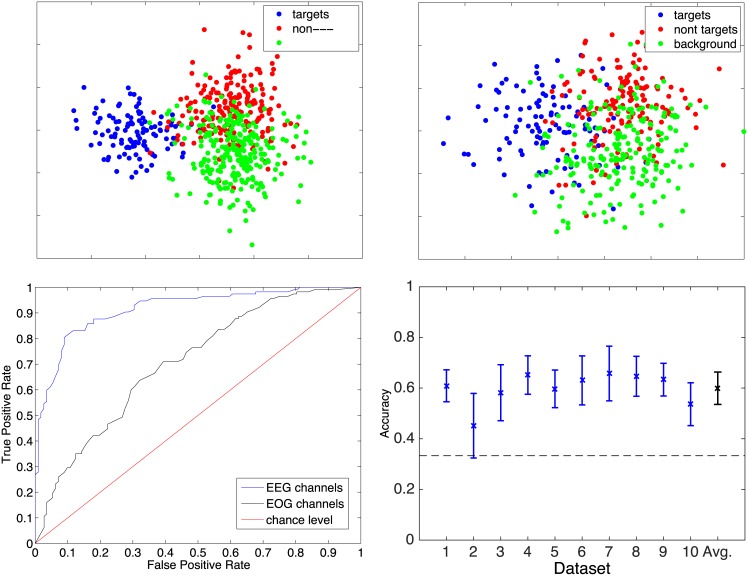
**Top row:** Verification of the linear separability for the best (left-) and poorest (right) datasets by inspecting the projections of the feature vectors on a 2d space spanned by the 2 largest eigenvectors of a 3-class FDA. The results illustrate that the 3-class FDA separates the data samples into three clearly distinguishable classes for the best dataset depending if the fixation appeared on targets, non-targets and background. Although the overlapping areas are larger in case of the poorest dataset, the three distinguishable classes are clearly evident. **Bottom left:** Comparison of the ROCs of participant 8 in the intra-subject classification scheme using only EEG channels and only EOG channels. The ROC values are averaged over the ten cross-validations runs. **Bottom right:** Classification results for the 3-class FDA. The dotted black line indicates the chance level.

The performance of the classifier was assessed using the area under curve (AUC) measure. The AUC is defined as the area under the so called “Receiver Operator Characteristics” (ROC) [35]. The ROC is obtained by mapping all real-valued classifier outputs *y*_*i*_ = ***x*** ⋅ ***w*** onto the interval [0, 1] and testing ${\hat{y}}_{i}=y_{i}+b_{k}$ for *b*_1_ = 0 and *b*_*K*_ = 1 with *k* = 100 and the *b*_*k*_ monotonically increasing and equally spaced. Then, the number of *true positives* is expressed as a function of the number of *false positives*. The AUC measure is chosen, because it is not effected by the prior class probability as is the simple measure of accuracy, which simply gives the ratio of correctly classified samples and is only meaningful when both classes are perfectly balanced in the full testset. For the AUC, the chance level is *always* AUC = 0.5. Please refer to Fig 2 (bottom left) as an illustrative example of the ROC of dataset 8 in the intra-subject classification scheme. A dataset corresponds to the data of one participant recorded in one continuous session.

## Results

In this section we report the results from the classification of the recorded EEG data. We grouped each dataset into three classes based on the fixation type the epoch corresponded to: target object, non-target object and blank background. In the following, we will use “target’, “non-target” and “background” to refer to these classes and the class labels, respectively. Additionally, we created a fourth, compound class made of the “non-target” and “background” class, which we denote as “rest”.

### ERP Analysis

**Fig 3:**
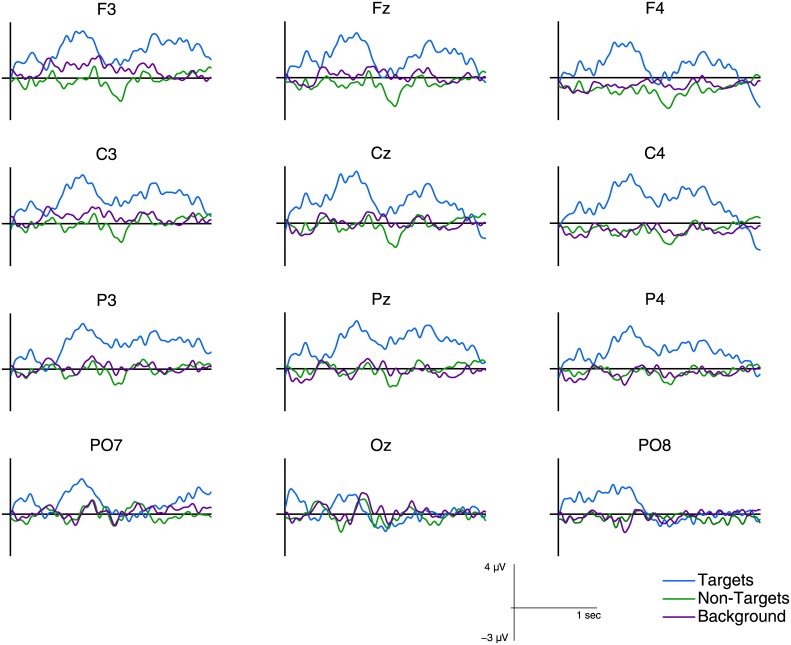
Grand average over all ten participants for target, non-target and background FRPs.

In a first step we analyzed the data by computing the standard grand average over all trials and all participants. The trials were grouped according to the three individual classes. Fig 3 shows the plots for the grand average FRPs for all twelve channels. One can observe a clear difference in the amplitude of the target average compared to the non-target and background average. The latter two conditions, in contrast, do not show clear differences. In the following, we will evaluate to which degree this differences are reflected when applying a classification approach on the *single-trial* data, instead of averaging over all trials.

### Linear separability

#### Procedure

The choice of an appropriate classifier includes considerations on the linear separability of the data. In P300-based BMI it is well known that linear classifiers provide an optimal performance and even outperform non-linear ones. In the past, we have performed extensive tests with linear (SVM with linear kernel, FDA) and non-linear (SVM with RBF kernel) classifiers to verify this assumption. Please refer to Kaper [36] for an in-depth elaboration of these comparisons. For the presented study, we additionally verified the linear separability of the data by inspecting the projections into a 2d space spanned by the 2 largest eigenvectors of a multi-class FDA.

#### Results

Fig 2 (top row) shows the projected data for the best and the poorest discriminable dataset. One can easily see that the target class samples (blue) are very well separated from those of the two other classes (i.e., non-target and background). The latter are also clustered in different regions of the plane. However, their linear separability is less clear due to a high degree of overlap. Nevertheless, this first analysis supports the notion that the classification of the data with a linear classifier should be tractable. The different degree of separability of the target class versus non-target as well as background, and further non-target versus background should be evaluated in more detail. To account for that, we started with a multi-class approach where the classifier represents the discrimination of all three classes in one model and then later proceeded with binary classifications of the following class distributions: target versus rest (non-target plus background), target versus non-target, target versus background, and non-target versus background.

### Intra-subject classification

#### Cross-validation procedure

EEG data typically exposes a high variability among people. Therefore, it is common to most BMI applications to classify the data in an intra-subject fashion. We followed this approach to ensure the best possible accuracy in the first place. Consequently, the dataset of each participant was divided into ten subsets. Each subset contained the same respective proportion of both classes than the total dataset. In the cross-validation nine out of the ten sets were merged to one training set and used to compute the PCA projection matrix and to train the FDA classifier. The classifier was subsequently used to classify the data of the tenth set (the test set). This procedure was iterated until each set has served once as a test set.

#### Three-class FDA results

We used the data from all three classes and a true 3-class FDA (not a 2—binary classifiers approach) to make a first evaluation. Here, we treated the datasets in the aforementioned intra-subject fashion (subject refers to one participant’s dataset recorded in a continuous session). A ten-fold cross-validation approach was used to ensure that training and testing data were strictly disjoint and that the variance of the classifier’s generalization capabilities could be appropriately assessed. We balanced the proportion of the three classes in the full set before splitting into the fold sets. Hence, we report here (and only here) the accuracy instead of the AUC, which is only defined for binary classification tasks. Extensions for multi-class problems exist, named the Volume under ROC surface. They are, however, used very rarely. The results of this approach are given in Fig 2 (bottom right). The plot depicts the mean accuracy and the standard deviation over the ten foldings per dataset. The dotted black line represents the chance level, which in case of three balanced classed is 0.33. The average accuracy over all ten datasets is 0.60 ± 0.06, which is considerably above chance.

#### Binary classification results

**Fig 4:**
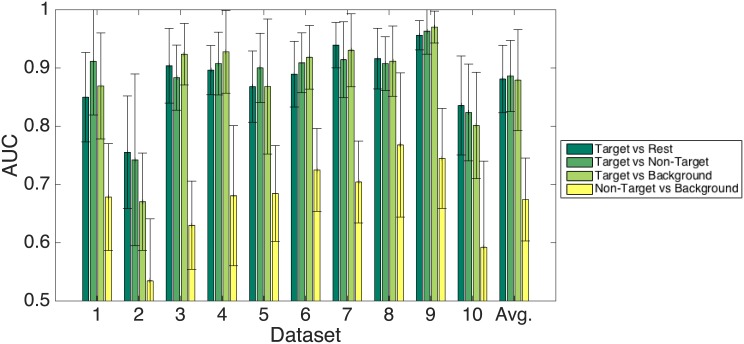
Classification results. Intra-subject 10-fold cross-validation with all 12 EEG channels. Please note that the y-axis starts at the chance level.

All results that are reported in this section refer to an AUC that is the average AUC over all ten cross-validation runs. Here, the classes were *not* balanced. The number of epochs per class represented the true prior distribution of the classes as it resulted from the experiment. Fig 4 depicts the results for all four binary classification tasks (see introduction to this section) that were evaluated in an intra-subject fashion. As could be expected from the results given in the previous paragraph, the target versus rest and correspondingly the target versus non-target and target versus background condition provides very good results. The average AUC over all participants is 0.88 ± 0.06, 0.89 ± 0.06, and 0.88 ± 0.09, respectively. Consistent with this expectation is the much lower AUC for the non-target versus background condition. The average AUC for this condition is 0.67 ± 0.07. A two-tailed, paired t-test on the latter and the target versus rest condition reveals that these differences are highly significant (*t* = 7.1, *p* ≪ 0.01).

### Inter-subject generalized classification

#### Procedure

We tested the generalization performance of the classifier across different participants (datasets). This is particularly interesting, because the intra-subject (and intra-session) approach requires a training dataset to be recorded prior to each session. An inter-subject generalized classifier would allow to use a brain interface right away, without additional effort to acquiring data and train a session-based classifier. We simulated such a scenario by training an FDA classifier on a compound set made of the data of nine out of the ten participants and tested the performance on the left-out set. Hence, the training dataset contained exclusively data from other participants.

#### Results

**Fig 5:**
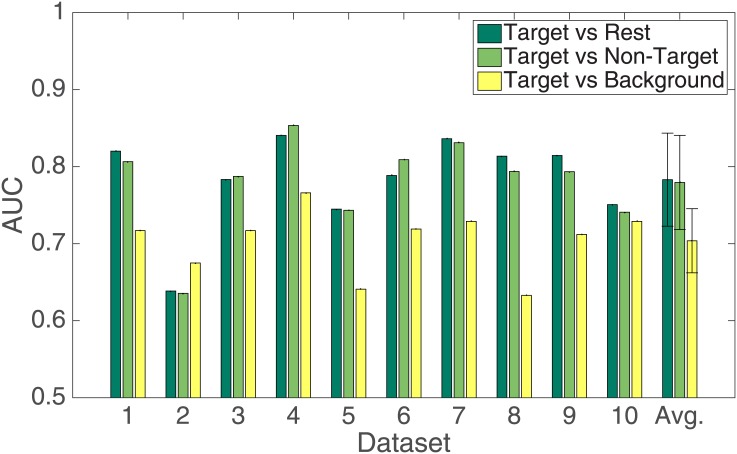
Classification results. Generalized inter-subject classifier using all 12 EEG channels. The classifier was trained on the compound data of nine participants and validated on the left-out set. The x-axis labels indicate the index of the test set. Please note that the y-axis starts at the chance level.

The AUC values of the inter-subject generalized classification approach are given in Fig 5. As expected, the classification results drop significantly for all datasets compared to the intra-subject scheme (two-tailed, paired t-test, *t* = 3.7, *p* < 0.01 for the results of the target versus rest condition). Nevertheless, the AUC is clearly above chance. The average AUC over all test sets is 0.78 in the target vs. all condition as well as in the target versus non-target condition. The target versus background condition yields only an AUC of 0.70. A two-tailed, paired t-test reveals that the latter condition is significantly different from the former two conditions (Target versus rest: *t* = 3.43, *p* = 0.003; Target versus Non-target: *t* = 3.24, *p* = 0.0045). This result is in contrast to the intra-subject approach, where the target versus background condition does not show significant differences compared to the other two conditions (*F* = 0.029, *p* = 0.97). The non-target versus background condition was not evaluated in this scheme because of the to-be-expected inferior results.

### EOG channel classification

#### Procedure

When classifying EEG data, it is of particular importance to ensure that the discriminative information contained in the data represents true neural activity and not merely eye movement artifacts. If the latter was the case, than the classification of the EOG data (that is, the four EOG channels that were recorded together with the EEG, see Methods section) should yield similar or even superior results. This would then mean that eye movements were sufficient to discriminate between target and non-target objects and the recording of EEG data did not provide additional value—what would be major objection to an FRP-based brain-machine interface. Therefore, we used EOG channel classification as a control condition to verify our claim that the relevant discriminative information corresponds to neural activity and is contained in the EEG data.

Consequently, we used the four EOG channels to build and test a classifier that relies on eye movements alone. The procedure for segmentation, feature extraction and classification equaled the intra-subject approach, only that all datasets contained solely the four EOG channels. The channels were used “as is” and not referenced to each other. We also tested using the two re-referenced VEOG and HEOG channels. The results, however, were inferior to those using the four raw channels.

#### Results

**Fig 6:**
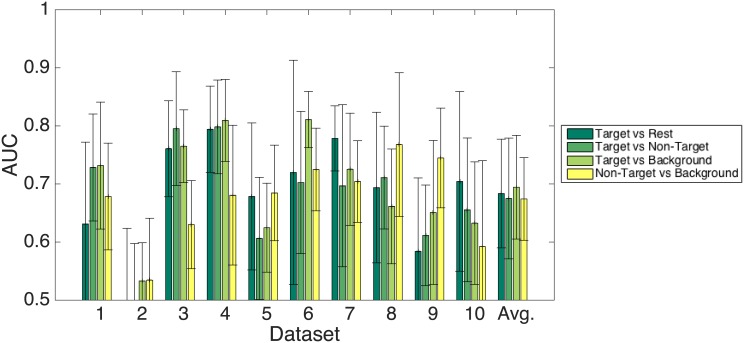
Classification results. Intra-subject 10-fold cross-validation with *only* the EOG channels. Please note that the y-axis starts at the chance level.

The results of the EOG classifier cross-validation are summarized in Fig 6. It is easy to see that the results are much lower than for the EEG data, although they are better than chance. The differences to the EEG classification are highly significant. In the target versus rest condition a two-tailed, paired t-test gives *t* = 5.67, *p* ≪ 0.01 (the three remaining conditions give almost identical results, with matching significance levels).

### Combined EEG and EOG channel classification

#### Procedure

In a final step, we combined the EEG and the EOG channels to form conjunct feature vectors. Our hypothesis was that if the relevant discriminative information is contained in the EEG data, the addition of the EOG channels should not improve the classification accuracy. Here, we used the full dataset with all recorded 16 channels—12 EEG and 4 EOG channels. All processing steps (filtering, segmentation, feature extraction, classification) were again identical to the intra-subject approach described earlier. This also holds for the cross-validation procedure. The difference here is that we tested only the target versus rest case.

#### Results

**Fig 7:**
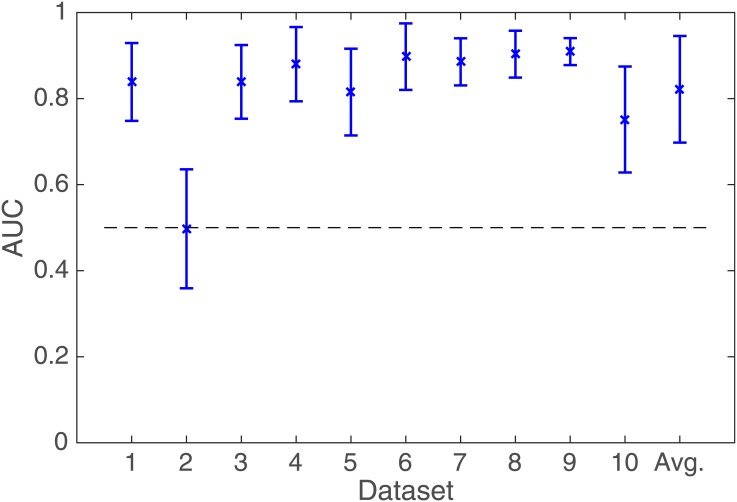
The results of classifying compound feature vectors with both the EEG and the EOG channels in the target versus rest case. The dotted line indicates the chance level.

Fig 7 shows the results of the combined EEG-EOG channel classification. The average AUC over all participants is 0.82 ± 0.12, compared to 0.88 ± 0.06 in the EEG only condition. Obviously, the former gives inferior results, however, the difference is not statistically significant (*t* = 1.37, *p* = 0.19). Nevertheless, it is clear that the addition of the EOG channel information does not improve the classification. This is consistent with the EOG-only case with its poor AUC. This validates once more the assumption that the relevant information for the discrimination task is contained in the EEG channels and thus truly originates from cortical activity and not from eye movements.

## Discussion

The results of the present study show that is possible to distinguish FRPs of target, non-target and background fixations with a very high accuracy. Thus, our study confirms and extends the results of Brouwer et al. [11]. In contrast to this previous work, we relieved several restrictions on the level of the stimuli as well as on the classification level. Firstly, our targets and non-targets were images of real world objects and not artificial symbols. Secondly, the task allowed free viewing. No particular search pattern was required and the participants could freely explore the scene. By using the gaze-contingent approach that revealed only a circular area of 3.5°of visual angle of the full stimulus at a time, we ensured that we had full control over the exact content of visual information that could be processed during a particular fixation. Hence, the process of aligning fixations, i.e. their onsets, to EEG potentials becomes more straightforward, reliable and less prone to errors resulting from uncertainties about the exact piece of visual information that triggered the particular ERP. This is beneficial for studying FRPs, because the occurrence of a potential can be exactly attributed to the relevant visual information such as a specific fixated object.

During data analysis, we set the temporal threshold for fixation detection to 60 ms (this value is recommended by the manufacturer of the eye tracker, LC Technology). This threshold is the minimal amount of time that the variation of the eye gaze coordinates remains below a spatial threshold. This value was chosen to ensure that always the first fixation onto an object is registered and no fixations are missed. This is important, because the first fixation is the one that is relevant to trigger the FRP. Typically, fixations onto objects are around 250 ms (which was also the case in our study, see Sec. “Fixation Detection”). However, the approach applied by the authors in [11] used only fixations with a duration longer than 500 ms. There, many valid fixations are not taken into account for the analysis. While the rationale for such long fixations, i.e., the minimization of eye artifacts in the aligned EEG data epoch, is comprehensible, this choice nevertheless disregards the typical properties of fixations in a natural viewing task. In practical applications, i.e. BMI systems, it is crucial to properly acknowledge the typical properties of the used modalities. Otherwise, one imposes unwanted and unnatural constraints on the modus operandi of the system. Therefore, offline studies like the one presented here should step by step relieve the constraints and evaluate an increasing degree of complexity toward real-world scenarios.

The classification procedure employed in this study used prior experience with P300-based BMI systems. Although typically the stimuli applied in BMI settings are much more structured and optimized for evoking the strongest possible cortical response, the classification task is rather similar. Our results suggest that this is a reasonable assumption. We were able to classify test data with accuracies significantly above chance, in both the intra-subject and the more demanding inter-subject generalized scheme. The latter is of particular importance for future real-time BMI applications based on the proposed approach. Every machine learning method, including the FDA classifier used in this study, depends on an amount of training data to learn the discriminative model. Applying an intra-subject scheme (which is the typical choice in BMI applications) requires to record a training dataset (in case of a supervised learning method including class labels) prior to using the system productively. Each user must therefore participate in a training session to provide the data for training the model. Thus, a ready-to-use BMI application, which is the desired case, needs to rely on an inter-subject generalized classification scheme. There, training data can be acquired from several users once and afterwards the system can be used right-away by an arbitrary number of people, without any additional preparation effort.

Our classification results are comparable to those typically achieved in P300 BMI settings. The latter fact indicates that FRPs elicited by the processing of target objects can be very well discriminated from the processing of non-target objects and a blank background, even on a single-trial level.

### Conclusion

Our results show that the detection of FRPs in a more complex and less constrained task is possible on a single-trial level. Furthermore, the classification accuracies that we obtained were significantly above chance and reached a level that corresponds to that typically expected in a P300-based BMI system. This is an important step toward applications exploiting FRPs in online systems. It opens an avenue for a whole range of applications beyond the “classical” BMI domain, which, to date, still mainly targets assistive technologies for patients with special, physical needs. ERPs, and consequently FRPs, convey a lot of valuable information on cognitive processes going on in the brain. Their excellent temporal resolution makes them an ideal candidate for extracting information on these processes in real-time. Furthermore, an FRP-based BMI promises to provide a novel means for intuitive human-machine interaction (HMI), for example, by providing cues to the machine which objects in a scene are relevant to the user, without the need to communicate that explicitly. Here, HMI can greatly benefit from the integration of eye tracking and EEG, because together these complementary modalities provide an implicit, holistic measure on ongoing cognitive processes.
